# Pathological and Practical Researches on Diseases of the Brain and the Spinal Cord

**Published:** 1828-03

**Authors:** 


### CRITICAL ANALYSES.

Quæ laudanda forent, et quæ culpanda, vicissim

Illa, prius, cretâ; mox hæc, carbone, *notamus*.—PERSIUS.

*Pathological and Practical Researches on Diseases of the Brain and the Spinal Cord.* By JOHN ABERCROMBIE, M.D. Fellow of the Royal College of Physicians of Edinburgh, &c.—8vo. pp. 444. Waugh and Innes, Edinburgh, 1828.

Dr. ABERCROMBIE has been long before the public as an industrious and successful cultivator of pathological knowledge, and his various contributions towards illustrating the characters and elucidating the nature of several perplexing forms of disease have acquired him a high reputation as a pathologist, wherever that essential branch of medical science is duly appreciated. The present volume embraces a more extended and connected view of a most important class of diseases, which formed the subject of a series of papers published by the author, some years ago, in the Edinburgh Medical and Surgical Journal. The plan followed in the course of the investigation is that now happily adopted by the best pathologists in their researches into the nature of diseases,—viz. a faithful record of facts and observations as they present themselves in the course of practice; and these not on a limited, but an extensive scale. The author disclaims all purpose of writing to support any particular system of doctrines, and sufficient evidence appears in the sequel to justify the assertion, and prove his readiness, at all times, to abandon theory and hypothesis when not supported by facts.

The Diseases of the Brain are arranged by the author under three heads:—1, The inflammatory affections; 2, the apoplectic affections; and, 3, the organic affections. A fourth part of the work is devoted to Diseases of the Spinal Cord and its membranes, with an appendix exhibiting a short outline of the Diseases of the Nerves.

In the first class, the author treats of inflammation of the dura mater,—of the arachnoid and pia mater, or *Meningitis*,—inflammation of the substance of the hemispheres,—and inflammation of the central parts and ventricles, comprehending the various forms of disease which have usually been included under the term *Acute Hydrocephalus*; with a short notice of certain conditions which appear to be allied in their nature to the inflammatory affections,—viz. tubercular disease of the brain, certain affections of the pericranium and bones of the cranium.

The consideration of the specific inflammations is prefaced by a general view of the symptoms which indicate inflammatory disease within the head. In this excellent and truly practical sketch, the author depicts not only those characters which mark the full development of disease, but also those more faint indications, and those combinations of symptoms, which denote the first invasion of this dangerous affection of the brain, justly considering an early diagnosis essential to the security of the patient and the reputation of the physician. It is only in the early stage of the affection that curative measures can be undertaken with a reasonable prospect of success; and, since to detect inflammation of the brain is to detect danger, the practitioner who is acquainted with those signs which foretell the coming mischief will be saved, in many cases, the mortification of giving a false prognosis.

The author acknowledges that, in the present state of our information on the subject, we are not able to say, with confidence, what symptoms indicate inflammation of the substance of the brain, as distinguished from inflammation of its membranes. In a practical point of view, perhaps, this difficulty is not of much importance; the practical requisite being the ability to detect the existence of this diseased action in any part within the head, particularly at an early stage of its progress. The symptoms on which we are to rely in forming our opinion appear under a variety of forms, depending probably on the seat of the inflammation, as well as on its degree of activity. The leading modifications our author refers to five heads:—

1. The *phrenitis* of systematic writers, sufficiently characterised by its nosological definition. This is seldom met with as an idiopathic disease, except when brought on by insolation and the abuse of strong liquors. As a symptomatic affection, it is met with in fever, mania, and after injuries of the head. It is generally fatal in the inflammatory stage, before the occurrence of the disorganization resulting from the inflammation, which in this case has usually its primary seat in the membranes of the brain. An affection, of not unfrequent occurrence, is referred to this head, which, from its being unattended with the high febrile excitement of phrenitis, is apt to be mistaken for mania, but differs from it in the material circumstance of its proving rapidly fatal. It is characterised by a peculiar aberration of mind, without any complaint of pain. There is great restlessness, quickness and impatience of manner, obstinate watchfulness, and incessant rapid talking, the patient rambling from one subject to another, with little connexion, but often without any actual hallucination. He knows those about him, and generally answers distinctly questions that are put to him. The chief morbid appearance on dissection is a highly vascular state of the pia mater.

2. In a second form, the first symptom that excites alarm is a sudden attack of general convulsions, either without any previous illness, or preceded by slight complaints which had attracted little notice. The convulsion may be long and severe, followed immediately by coma, terminating fatally in a few days; or it may occur frequently, the patient in the interval being sensible; but, after the lapse of twelve or twenty-four hours, passes into coma. From this state the patient may appear to recover, and to go on favorably for several days, when, without any warning, the convulsions return, and terminate in fatal coma. In some cases the convulsion is confined to one side of the body or to one limb, and is usually followed by partial paralysis; and in others the first symptom is an attack of paralysis, bearing a close resemblance to the ordinary attack of hemiplegia. This form of disease, in which the prominent symptoms are spasmodic or paralytic, is usually connected with inflammation of a defined part of the cerebral substance passing into suppuration or ramollissement, though it may be fatal without passing beyond the state of simple inflammation. In other cases, again, the inflammation is confined to the membranes.

3. The third class of symptoms indicative of inflammation within the head, is mostly observed in children, but may also appear in adults. In this instance the affection does not invade with the peculiar violence observable in the two former varieties. It is usually preceded by a day or two of languor and peevishness, followed by an accession of fever, which is sometimes ushered in by severe shivering. The patient is oppressed, and unwilling to be disturbed, and complains of acute pain in some part of the head, with flushing of the face and impatience of light, disturbed sleep, frightful dreams, and violent grinding of the teeth. These symptoms may occur with disorder of the alimentary canal, but not unfrequently when the bowels are natural and the tongue clean. After some days, slight delirium begins to appear, at first transient and only during the night. In other cases, instead of delirium, there is a peculiar forgetfulness, the patient using one word instead of another, misnaming things, mistaking the day or the time of the day ; a confusion of thought of which he seems sensible, and anxious to divest himself. These symptoms are followed by drowsiness, which soon passes into coma, the more active symptoms at the same time abating.

The pulse, from being frequent, falls to the natural standard, or below it; the acute sensibility to light gives place to dulness of the eyes, squinting, or double vision; and, lastly, dilatation of the pupil and blindness. After a day or two, or perhaps a few hours only, the pulse begins to rise again, and rises to extreme frequency; the stupor continuing at the same time to increase till it becomes perfect coma, in which the patient lies for a few days before death closes the scene. There is an extreme inequality in the frequency of the pulse, varying perhaps every minute, or every time it is counted. This remarkable inequality the author deems deserving of particular attention, as being peculiar to dangerous affections of the head. In some cases the pulse does not come down, but continues uniformly frequent throughout the whole course of the disease. The duration of the complaint is extremely various. It is sometimes protracted to three weeks, and in others, particularly in young children, it proves fatal in five or six days. At some period of the disease, generally when the pulse is falling in frequency, or when it is beginning to rise after becoming slow, there is a remarkable remission of all the symptoms, giving sanguine but deceitful hopes of recovery.

4. The fourth modification of symptoms usually occurs in young persons, towards the age of puberty and upwards. The affection begins like a slight feverish disorder, with headache, and for a long time excites no alarm. The febrile symptoms are slight, and may yield to the usual treatment, the tongue becoming cleaner, and the appetite better; but the headache continues obstinate, seldom severe, but never quite gone, and attended with a listlessness and oppression which cannot be accounted for by the degree of fever present. In this way the disease may go on for several days, until, about the twelfth or fourteenth day, the pulse suddenly falls to the natural standard, or below it, while the headache increases, with an evident tendency to stupor. This instantly marks a head affection of the most dangerous character; stupor and coma supervene; the pulse rises, and the disease runs the fatal career described as marking the progress of the last variety, to which it bears a close resemblance, but differs from it in the very insidious mode of its encroachment; cases occasionally occurring in which there is not even the slightest complaint of headache through the whole course of the disease.

5. Another form of the disease, which is most commonly met with in adults, begins with violent pain in the head, generally deep-seated, *without fever*. The patient is found lying in bed, with a look of much oppression, holding his head with both hands, and unwilling to be disturbed, or tossing about from the violence of the pain. The face is sometimes flushed, at other times pale, with a contracted pupil and impatience of light. The pulse, which at first was at the natural standard, or was falling to fifty or sixty, rises in the progress of the complaint to 120 or 130. There is more or less delirium, which begins early, and that peculiar forgetfulness and confusion of thought already mentioned as characterising, in the absence of fever, dangerous cerebral affection. There is generally towards the end more or less also of coma, which usually sets in in five or six days, and continues to the last, but occasionally disappears before the fatal termination.

To the foregoing account of the most common forms which inflammatory affections of the brain assume, the author subjoins a simple enumeration of all the symptoms, as observed in the *head,* the *circulation,* the *muscles,* the *organs of sense,* and the *intellectual faculties;* but adds, that a minute attention to the *correspondence* of the symptoms is of more importance in establishing a diagnosis than any individual symptom, however strongly marked. Thus, the peculiar oppression, the headache, and delirium, which accompany high fever, are not reckoned particularly unfavorable; while the same symptoms occurring without fever, or with very slight fever, would indicate a head affection of the most dangerous character. This truth is fully established by the cases detailed in illustration of the text; and in the following extract, as well as in other places, we find the author anxious to impress on practitioners the necessity, in all head affections, of narrowly watching the case long after symptoms of a serious or suspicious tendency have subsided.

"In all the forms of this dangerous affection, there is great variety in the symptoms, and much observation is required to put us fully upon our guard against the insidious characters which many of the cases assume, and the deceitful appearances of amendment which often take place in all the forms of the disease. Even in those cases which have assumed the most formidable aspect, every alarming symptom may subside. The pulse perhaps continues frequent, but it also is coming down; at our successive visits we find it falling regularly, and we are disposed to hope that a few days will bring the case to a favorable termination. During this deceitful interval, which may continue for several days, I have known a parent intimate to the medical attendant that his farther visits were unnecessary; and I have known a physician take his leave, considering his patient as convalescent. As the pulse falls, the patient is disposed to sleep: this perhaps is considered as favorable; it falls to the natural standard; he then sleeps almost constantly, and in another day this sleep terminates in coma. The pulse then begins to rise again; it rises to extreme frequency; and in a few days more the patient dies. All this may go on with very little complaint of headache, and without any symptom that will lead a superficial observer to suspect danger, until he finds his patient gliding into coma at the very time when he expects recovery; for the period when the pulse falls to the natural standard is the time when the coma becomes evident, and the situation of the patient probably hopeless. Whenever, therefore, at any period of a febrile disease, there have been remarkable symptoms in the head, such as violent headache, with vomiting and impatience of light, stupor, convulsive affections, or affections of the sight, though these symptoms may have entirely subsided, and the complaint may have again assumed the characters of simple fever, we must not consider the danger as over, but must be upon our guard against a period of anxiety which is still before us. An attentive observer may generally remark, in such cases, something which leads him to suspect that the appearance of amendment is deceitful. Sometimes there is a dilated state of the pupil, giving to the eye a peculiar expression, and sometimes there is a remarkable tendency to sleep. Frequently something unusual may be observed in the patient's manner, such as a fretfulness or querulousness which is not natural to him; a quick and hurried manner of speaking, or, on the contrary, a remarkable slowness of speech; difficult articulation, or a peculiar confusion of thought and forgetfulness on particular subjects. But it cannot be too strongly impressed upon the younger part of the profession, that cases occur in which all these symptoms are wanting, and in which the patient appears for several days to be in the most hopeful state of recovery, while in fact his disease is advancing rapidly to a fatal termination." (P. 13.)

Besides the modifications described in the above outline, numerous varieties occur which it is not easy to include under any general arrangement. All of them probably depend on the seat of the disease, the result of the morbid action, and its degree of intensity. The latter we find varying from the pure scrofulous inflammation, with the lowest degree of activity, to that accompanied with symptoms of the highest excitement.

When we investigate these affections in a pathological point of view, we find them terminating fatally in various ways. They may prove fatal in the first stage without any actual result of inflammation; and this, we have seen, may occur whether the inflammation is seated in the substance of the brain or in the membranes. The more common terminations, however, are by serous effusion, deposition of false membranes, suppuration, ramollissement, and lastly in a chronic form, with thickening of the membranes, contractions and obliteration of the sinuses, or caries of the bones. But the results obtained on dissection are not always uniform, the terminations being variously combined, but all of them generally accompanied with more or less of serous effusion.

We find *serous effusion* varying in its seat, as well as in its quality and quantity. In most instances it may be traced to direct inflammatory action, though there is reason to believe that, as in other parts of the body, it may be the result of obstructed circulation in the veins, such as might be produced by congestion, the pressure of tumors, and chronic disease of the sinuses. It is found in the ventricles, under the arachnoid, betwixt the arachnoid and dura mater, and betwixt the latter and the bone. In quality it may be limpid, turbid, flocculent, or even bloody and semipurulent; and in quantity, it varies from a few drachms to eight or ten ounces.

The deposition of false membranes is the result of inflammation of the membranous parts, and may be met with wherever these exist, though its most common seat is under the arachnoid; in other cases, it follows the course of the pia mater, producing complete adhesion of the convolutions to each other.

*Suppuration* takes place under various forms: sometimes as a thin uniform layer under the arachnoid, or between the different membranes; occasionally in the ventricles, but most commonly as well-defined encysted abscesses in the substance of the brain itself. Sometimes these abscesses are extremely minute: an example is given of a well-defined abscess, no larger than a common bean, in the substance of the corpus striatum. At other times a considerable portion of the cerebral substance is found in a broken-down state, in which, without any well-defined cavity, pus is found mixed with disorganised cerebral matter.

The morbid condition of the brain which has received the name of softening, or *ramollissement,* has given rise to some difference of opinion as to its precise nature. It consists in a conversion of a part of the cerebral substance into a soft pulpy mass, retaining its natural colour, but having lost its cohesion and consistence, yet differing from suppuration in having neither the colour nor the fœtor of pus. Since the time when our author first investigated this condition of the brain, and ranked it as a result of inflammation, it has been the subject of much research by M. Rostan and other French pathologists, who contend for a different nature of the affection, believing it to be a disease *sui generis*,—a peculiar and primary disease of the brain, though occasionally a consequence of inflammation. Dr. Abercrombie endeavours to reconcile this diversity of opinion on the principle that this peculiar softening of the brain is analogous to gangrene in other parts of the body, and that, like gangrene, it may arise under two conditions of the system,—viz. over-action, in one case, and failure of the circulation, from disease of the arteries, in the other. In the cases related by M. Rostan, this disorganization was met with chiefly in very old people, and in connexion with attacks of an apoplectic or paralytic kind. In those which fell under our author's observation, it occurred chiefly in young persons, in connexion with cerebral affections of an acute character, and was seated, for the most part, in the dense central parts of the brain, and frequently combined with appearances of inflammation, or the results of inflammatory action, as suppuration, effusion, and the deposition of false membranes. It is found in combination with effusion in a very large proportion of the ordinary cases of hydrocephalus, and is sometimes the only morbid appearance met with after death in exquisitely marked cases of this affection. From this comparative review of the circumstances under which the affection occurred to M. Rostan and to himself, the author is disposed to consider that described by the French pathologists as the consequence of languor or failure of the circulation, and the examples which fell under his own observation to an opposite cause, increased vascular action, or active inflammation.

From the consideration of the symptoms and terminations in general of inflammatory affections of the brain and its envelopes, the author passes to a detail of cases illustrative of the disease as seated in particular textures: the different membranes, the ventricles, and various parts of the substance of the brain.

Inflammation of the dura mater is seldom met with as an idiopathic affection. One case, however, is detailed in which it appears to have been the primary disease, though in its progress it became complicated with extensive inflammation of the arachnoid. But it is frequently met with in another form, in which it occurs in connexion with affections of the ear, and of the petrous portion of the temporal bone. It is a highly dangerous affection, from the insidious way in which it invades, the first symptom being pain in the ear, which is usually treated lightly, till the supervention of shivering, drowsiness, oppression, and perhaps slight delirium, direct attention to its true character. Great restlessness follows, ending speedily in coma and death. A discharge of matter from the ear may precede or follow the serious symptoms, or these may supervene on a sudden suppression of the discharge. On dissection, we generally find caries of the temporal bone, with a corresponding portion of the dura mater detached, thickened, or ulcerated. In some cases there is a superficial abscess of the brain itself, or effusion into the ventricles, with other evidences of general cerebral affection. In one case detailed by the author, the left lateral sinus was remarkably diseased through its whole extent, and nearly obliterated. In most of the cases, the patients had been subject to attacks of pain in the ear, with discharge of matter. Sometimes the coma and other alarming symptoms are suddenly relieved by a discharge of purulent matter from the ear, but the relief is generally but temporary; and, in those cases in which complete recovery follows, it is by no means certain that the discharge comes from the cavity of the cranium, for there is reason to believe that an accumulation of matter within the tympanum will give rise to symptoms of oppression. A case of this kind is described by ITARD, in which partial alleviation of urgent symptoms took place from matter escaping by the eustachian tube, and complete relief followed from puncturing the membrana tympani.

Inflammation of the dura mater, with its consequences, is also found connected with disease in the nose. Cases of this kind are mentioned by BONETUS, LIEUTAUD, and MORGAGNI, in which persons liable to pain in the forehead, with purulent discharge from the nose, become at last forgetful, delirious, and die comatose. On dissection, the ethmoid bone is found carious, the dura mater corresponding to it diseased, with deposition of pus, and perhaps abscess of the adjoining portion of the brain.

Under the term *Meningitis*, the author treats of inflammation of the arachnoid and pia mater together, as it is very difficult, and perhaps of no material importance in practice, to discriminate between them. The disease usually terminates in a deposition of false membrane betwixt the arachnoid and pia mater, spreading uniformly over the surface of the convolutions, or dipping down between them and gluing them together. This deposition is met with most abundantly in the sulci between the convolutions, and in the depressions between the lobes, and often, of a gelatinous consistence, about the optic nerves. The membranes themselves, particularly the pia mater, often exhibit an intense degree of vascularity.

Several cases are given in illustration of this affection, exhibiting remarkable diversity of symptoms. The most usual form of attack is by convulsion, generally severe and long continued, and passing into coma, which afterwards alternates only with a repetition of the convulsions till the case terminates fatally. In other cases which run a fatal course, there is a remarkable remission of the symptoms at some period of the disease. A degree of this affection often attends other cerebral inflammations, but it is often met with entirely uncombined. The convulsive affections of children, which are apt to be indiscriminately attributed to dentition, are probably not unfrequently connected with this affection. An important variety is described, and two cases detailed, in which, from the prominent character of some of the symptoms, the affection is apt to be mistaken for mania, or, in females, for a modification of hysteria. The following is our author's account of this dangerous modification:

"Another important modification of the disease occurs in an insidious and highly dangerous affection, which I think has been little attended to by writers on the diseases of the brain. It is apt to be mistaken for mania, or, in females, for a modification of hysteria; and in this manner the dangerous nature of it has sometimes been overlooked, until it proved rapidly and unexpectedly fatal. It sometimes commences with depression of spirits, which after a short time passes off very suddenly, and is at once succeeded by an unusual degree of cheerfulness, which is rapidly followed by maniacal excitement. In other cases, these preliminary stages are less remarkable, the affection, when it first excites attention, being in its more confirmed form. This is in general distinguished by remarkable quickness of manner, rapid incessant talking, and rambling from one subject to another, with obstinate watchfulness, and a small frequent pulse. Sometimes there is hallucination, or conception of persons or things which are not present; but in others this is entirely wanting. The progress of the affection is generally rapid: in some cases it passes into convulsion and coma; but in general it is fatal by a sudden sinking of the vital powers, supervening upon the high excitement, without coma. The principal morbid appearance is a highly vascular state of the pia mater, sometimes with very slight effusion betwixt it and the arachnoid. The disease is one of extreme danger, and does not in general admit of very active treatment. General bleeding is not well borne, and the treatment must in general be confined to topical bleeding with purgatives, antimonials, and the powerful application of cold to the head. The affection is most common in females of a delicate irritable habit, but also occurs in males, especially in those who have been addicted to intemperance. I have, however, seen it in one case, in a gentleman between forty and fifty, of stout make and very temperate habits. The cause of death is obscure: it seems in general to be a sudden sinking of the vital powers, supervening upon the high excitement, without any of the actual results of inflammation." (P. 62.)

Meningitis appears also in a chronic form, exhibiting a protracted succession of symptoms,—chiefly headache, impaired vision, partial paralysis, convulsions or maniacal symptoms, and presenting, on dissection, thickening of the membranes, and depositions apparently of long standing.

*Inflammation of the Hemispheres.*—The usual terminations of inflammation seated in the substance of the brain composing the hemispheres, are ramollissement and suppuration: the latter occurring in an undefined form, or as an abscess regularly encysted; or, lastly, in the form of ulceration of the surface of the brain. It may also prove fatal in the inflammatory stage, before any of these results have taken place.

Numerous highly interesting cases, illustrative of this pathological condition of the brain, are detailed by the author; but, from want of uniformity in the symptoms, equal to, if not greater than what attends other cerebral inflammations, it is not possible to advance any general statement of the phenomena which attend it, or to refer particular symptoms to the particular seats or terminations of the disease. It is not, in general, attended by the high febrile excitement present in inflammation of the membranes. The most constant symptoms are pain in the head, often deep seated, and referred to a particular part, ending in coma, or in convulsions of one or more limbs, and followed by paralysis of the affected parts. The paralysis may occur early, and be only transient, or at a later stage of the disease, and prove permanent, when it may be presumed that the inflammation has terminated in ramollissement or suppuration. At the commencement we also find embarrassment of speech, obtuseness of hearing, and impaired vision, passing into complete loss of speech, blindness, and other characters of profound coma. Sometimes a remarkable recovery of speech takes place in the course of the disease, as well as a remission, under active treatment, of other characteristic symptoms. Inflammation of the hemispheres occurs also in a chronic form, proving fatal with or without the usual results—ramollissement and suppuration.

(To be continued.)

